# Moving Beyond Medical Statistics: A Systematic Review on Missing Data Handling in Electronic Health Records

**DOI:** 10.34133/hds.0176

**Published:** 2024-12-04

**Authors:** Wenhui Ren, Zheng Liu, Yanqiu Wu, Zhilong Zhang, Shenda Hong, Huixin Liu, on behalf of the Missing Data in Electronic health Records (MINDER) Group

**Affiliations:** ^1^ Department of Clinical Epidemiology and Biostatistics, Peking University People’s Hospital, Beijing, China.; ^2^ National Institute of Health Data Science, Peking University, Beijing, China; ^3^ Institute of Medical Technology, Health Science Center of Peking University, Beijing, China.

## Abstract

**Background:** Missing data in electronic health records (EHRs) presents significant challenges in medical studies. Many methods have been proposed, but uncertainty exists regarding the current state of missing data addressing methods applied for EHR and which strategy performs better within specific contexts. **Methods:** All studies referencing EHR and missing data methods published from their inception until 2024 March 30 were searched via the MEDLINE, EMBASE, and Digital Bibliography and Library Project databases. The characteristics of the included studies were extracted. We also compared the performance of various methods under different missingness scenarios. **Results:** After screening, 46 studies published between 2010 and 2024 were included. Three missingness mechanisms were simulated when evaluating the missing data methods: missing completely at random (29/46), missing at random (20/46), and missing not at random (21/46). Multiple imputation by chained equations (MICE) was the most popular statistical method, whereas generative adversarial network-based methods and the k nearest neighbor (KNN) classification were the common deep-learning-based or traditional machine-learning-based methods, respectively. Among the 26 articles comparing the performance among medical statistical and machine learning approaches, traditional machine learning or deep learning methods generally outperformed statistical methods. Med.KNN and context-aware time-series imputation performed better for longitudinal datasets, whereas probabilistic principal component analysis and MICE-based methods were optimal for cross-sectional datasets. **Conclusions:** Machine learning methods show significant promise for addressing missing data in EHRs. However, no single approach provides a universally generalizable solution. Standardized benchmarking analyses are essential to evaluate these methods across different missingness scenarios.

## Introduction

Approximately 95% of hospitals in the United States and 85.3% in China use electronic health record (EHR) systems [1,2]. Because EHRs are accumulating increasingly rich real-world clinical data generated during routine medical practice, there is a growing desire in academic and professional circles to use these data in medical research. The dynamic demographic, prognostic, and empirical data extracted from EHRs have been extensively applied in clinical trials [3], treatment effectiveness evaluations [4], clinical prediction model development [5], postmarketing drug surveillance [6], and genetic association studies [7]. However, missing data in EHRs presents a major challenge in the application of EHRs in healthcare-related studies [8].

In 1976, Rubin formalized the concept of missingness mechanisms [9], including missing completely at random (MCAR), missing at random (MAR), and missing not at random (MNAR). A method that is best suitable for mitigating the bias caused by missing data remains unknown, and several models have been proposed based on these 3 mechanisms. One intuitive approach is to fill in the missing values and then perform the subsequent analysis. An example is multiple imputation (MI), the most popular missing data handling method used in medical statistics [10]. Notably, several machine-learning-based methods have shown impressive results in this area, providing greater statistical power and less biased estimations [11,12].

Although many methods have been proposed to address this issue [13–15], uncertainty exists regarding how well they perform in multiple contexts. Published research on missing data processing strategies has not extensively examined the utilization of EHR datasets [16,17]. Furthermore, prior systematic reviews have focused on the presence of missing data in EHRs or are limited to specific types of missing data handling methods (i.e., imputation) [18–20]. Mitigation requires a thorough and valid benchmark evaluation that assesses the performance of missing data methods in EHRs across multiple missing scenarios. As such, this review summarizes published research on EHR-based missing data methods and provides strategic recommendations for given missing scenarios.

## Methods

This systematic review is reported in accordance with the Preferred Reporting Items for Systematic Reviews and Meta-Analysis (PRISMA) statement (see Supplementary Materials 1).

### Inclusion and exclusion criteria

The following eligibility criteria were applied: (a) English-language articles, (b) original published articles, and (c) studies that referenced EHR datasets and evaluated the performance of missing data handling methods. Exclusion criteria included the following: (a) reviews, (b) studies not applying missing EHR data, and (c) papers for which full-text versions were unavailable.

### Literature search

The search strategy was designed for MEDLINE, EMBASE, and Digital Bibliography and Library Project from their inception until 2024 March 30, using variations of search terms including “electronic health records”, “missing data/data imputation”, and “method/approach/strategy” (see Supplementary Materials 2). We meticulously examined the references cited in the obtained articles (i.e., snowballing) to identify additional relevant research.

### Study selection

**Fig. 1.**
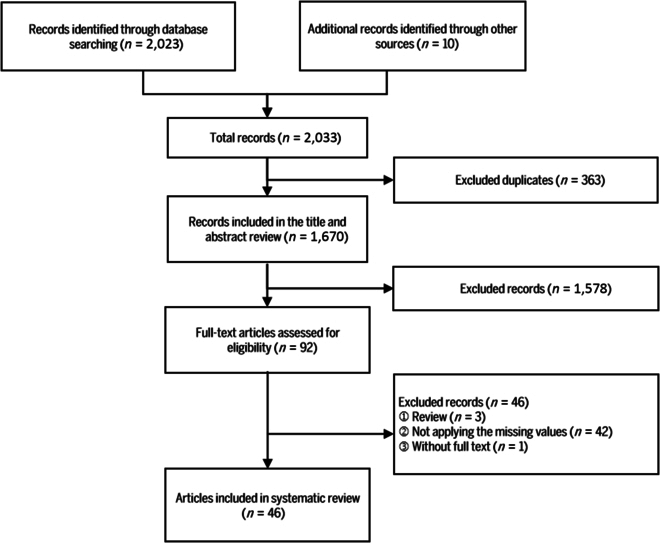
PRISMA systematic review flow diagram. Search databases included MEDLINE, EMBASE, and Digital Bibliography & Library Project.

The flow diagram of the PRISMA study is depicted in Fig. 1

. Two reviewers (H.X.L. and W.H.R.) independently reviewed the titles and abstracts of the papers. Discrepancies were resolved through discussions and subsequent full-text screenings.

### Data extraction

We developed a standardized data extraction template that cataloged basic publication information (i.e., publication year, study design, and the name of datasets), missingness scenarios (i.e., missingness mechanisms, percentage of missing values, and missingness patterns [i.e., monotone and arbitrary types]), and type of missing data (i.e., covariates and outcomes). Missing data handling methods were also extracted and divided into deep learning, traditional machine learning, and medical statistical methods according to the main architectures of different methods [16,18].

### Data analysis

To compare the effectiveness of various methods for addressing missing data, we classified the missingness scenarios for datasets with different study designs (cross-sectional study or longitudinal study), sample size (<10,000 or ≥10,000), percentages of missing values (≤50% or ≥50%), and missingness mechanisms (MCAR, MAR, and MNAR). Given the different purposes and evaluation indicators in various studies (imputation quality: RMSE, SE, and bias; downstream performance: AUC, F1-score, and OR/HR), we mainly evaluated the performance of different methods according to the results reported in the original included studies.

## Results

### Characteristics of the included studies

A total of 2,033 articles were identified in the preliminary literature search. After removing duplicate entries and applying the inclusion and exclusion criteria, 46 articles were selected for further review [8,11,13–15,21–61] (Fig. 1). Table 1 presents the characteristics of these articles, published between 2010 and 2024. Most studies (31/46) focused on missing longitudinal data, primarily from open-source datasets such as the Medical Information Mart for Intensive Care (MIMIC) (*n* = 12), the UC Irvine Machine Learning Repository (UCI) (*n* = 8), and the Health Improvement Network (THIN) (*n* = 2). The simulated percentage of missing values among the included studies ranged from 1% to 99%.

**Table 1.** Characteristics of 46 included studies

Author	Year	Data types	Datasets^a^ [sample size]
Weng et al. [51]	2024	Longitudinal data	MIMIC IV [*n* = 4,849]
Psychogyios et al. [56]	2023	Longitudinal data; cross-sectional data	Framingham Heart Study [*n* = 4,434]; Stroke dataset [*n* = 5,110]; Physionet heart failure [*n* = 2,008]; UCI heart disease [*n* = 303]
Zhou et al. [53]	2023	NR	Simulated datasets [*n* = 1,000]
Sisk et al. [54]	2023	Longitudinal data	Simulated datasets [*n* ≤ 1,000]; MIMIC-III [>60,000]
Kazijevs and Samad [14]	2023	Longitudinal data	DACMI [*n* = 8,266]; sepsis [*n* = 2,164]; hypotension [*n* = 3,910]; IEEEPPG [*n* = 3,096]; heartbeat [*n* = 409]
Bernardini et al. [11]	2023	Longitudinal data	MDC dataset [*n* = 40,555]; MIMIC-III dataset [*n* = 8,509]
Shadbahr et al. [21]	2023	Longitudinal data	1. Three real world datasets: breast cancer [*n* = 1,756]; MIMIC-III [*n* = 7,214]; NHSX COVID-19 [*n* = 851]
			2. Two simulated datasets: simulated (N) [*n* = 1,000]; simulated (N;C) [*n* = 1,000]
Li et al. [22]	2023	Longitudinal data	MIMIC-III [*n* = 46,288]; MIMIC-IV [*n* = 366,504]
Vader et al. [23]	2023	Longitudinal data	Advanced Bladder Cancer EHR Data [*n* = 4,361]
Getz et al. [24]	2023	Longitudinal data	A nationwide EHR-derived deidentified database for patients with metastatic urothelial carcinoma [*n* = 6,102]
Ferri et al. [25]	2023	Longitudinal data	Patients at emergency admission with SARS-CoV-2 infection from Spain [*n* = 35,411]
Shen et al. [57]	2022	Longitudinal data	Simulated datasets [*n* = NA]; EHR dataset from Mayo Clinic [*n* = 37,488]
Perez-Lebel et al. [15]	2022	Longitudinal data	Traumabase [*n* > 20,000]; UK Biobank [*n* > 500,000]; MIMIC-III [*n* = 60,000]; NHIS [*n* = 87,500]
Samad et al. [27]	2022	Cross-sectional data	Breast cancer [*n* = 569]; dermatology [*n* = 358]; SkillCraft1 master table [*n* = 3,395]; wine quality [*n* = 4,898]; default of credit card clients [*n* = 30,000]; mice protein expression [*n* = 552]
Pereira et al. [26]	2022	NR	34 Public datasets from the medical context; cover different pathologies and domains of clinical research based on routinely collected healthcare data [*n*: range from 68 to 2,126]; a heart failure dataset for downstream classification task evaluation [*n* = 2,008]
Paul et al. [28]	2022	Longitudinal data	The Centricity Electronic Medical Records [minimum 12-month treatment duration *n* = 47,460; minimum 24-month treatment duration *n* = 29,171]
Mulyadi et al. [29]	2022	Longitudinal data	PhysioNet 2012 challenge [*n* = 4,000]; MIMIC III [*n* = 13,998]
Ouyang et al. [58]	2021	Longitudinal data	PhysioNet 2012 challenge [*n* = 4,000]; MIMIC III [*n* = 11,869]
Lim et al. [59]	2021	Longitudinal data	1. Six UCI Machine Learning Datasets (banknote [*n* = 1,372]; concrete [*n* = 1,030]; hepmass [*n* = 300,000]; power [*n* = 1,000,000]; red [*n* = 1,599]; white [*n* = 4,898]); Physionet 2012 Challenge Dataset [*n* = 12,000]
			2. The simulated datasets [*n* = 100,000]
Zhou and Saghapour [52]	2021	Longitudinal data	MIMIC III [*n* > 40,000]; UCI Machine Learning Repository: Boston [*n* = NA]; spam [*n* = 4,601]; letter [*n* = 20,000]; breast cancer [*n* = 569]
Bertsimas et al. [30]	2021	Longitudinal data	1. Three real-world clinical datasets: FHS dataset [*n* = 5,209]; DFCI dataset [*n* = 3,228]; PPMI dataset [*n* = 338]
			2. One simulated dataset [*n* = 10,000]
Mikalsen et al. [31]	2021	Longitudinal data	1. Four MTS benchmark datasets from the UCR and UCI databases: uWave [*n* = 4,478]; Char.Traj. [*n* = 2,858]; Wafer [*n* = 1,194]; Japan.vow [*n* = 640]
			2. Electronic health records for all patients that underwent a gastrointestinal surgical procedure at University Hospital of North Norway [*n* = 7,741]
			3. A synthetic MTS dataset [*n* = NA]
Jager et al. [13]	2021	NR	69 datasets [*n* = 3,000 to 100,000 observations]
Li et al. [8]	2021	Longitudinal data	Two distinct datasets were used: the GNSIS cohort [*n* = 9,037]; the Sutter Health heart failure cohort [*n* = 5,192]
Gwon et al. [32]	2021	NR	CardioNet (a real-world electronic medical record) [selected 10,000 of them as a teacher data and 50,000 of them as a student data. 10,000 teachers are complete data without missing values; and 50,000 students contain missing values]
Jazayeri et al. [60]	2020	Longitudinal data	MIMIC III [*n* = 8,267]
Zhang et al. [61]	2020	Longitudinal data	EHRs collected from the clinics in New York City [*n* = 2,267]
Leyrat et al. [55]	2020	Cross-sectional data	The simulated datasets [*n* = 500/1,000]
Xu et al. [33]	2020	Longitudinal data	A sample of patients diagnosed with ischemic heart disease at a major healthcare organization in Taiwan [*n* = 5,156]
Venugopalan et al. [50]	2019	Longitudinal data	MIMIC II [*n* = 32,331]
Nagarajan et al. [34]	2019	NR	Ten biomedical classification data sets downloaded from Kaggle and UCI data repository [*n* = 4,610]
Hegde et al. [35]	2019	Cross-sectional data	MCHS enterprise data warehouse [*n* = 696]
Pham et al. [36]	2019	NR	THIN databases [*n* < 12,000,000]; simulated dataset [*n* = 5,000]
Beaulieu-Jones et al. [49]	2018	Cross-sectional data	Outpatient laboratory results [*n* = 602,366]
Yoon et al. [37]	2018	NR	Five real-world datasets from UCI Machine Learning Repository: breast [*n* = 569]; spam [*n* = 4,601]; letter [*n* = 20,000]; credit [*n* = 30,000]; and news [*n* = 39,797]
Martín-Merino et al. [38]	2018	Longitudinal data	BIFAP [*n* = 95,057]; the EpiChron Cohort [*n* = 12,688]; CPRD [*n* = 161,202]
Luo et al. [39]	2018	Longitudinal data	A dataset from the Massachusetts General Hospital about inpatient test results for the 13 analytes [*n* included 313,0501 test results across 266,112 patient collections and 19,008 unique hospital admissions]
Beaulieu-Jones and Moore [40]	2017	Longitudinal data	PRO-ACT [*n* = 1,824]
Razzaghi et al. [41]	2016	Cross-sectional data	1. Public (UCI and the cod-rna dataset) dataset
			2. Healthcare datasets (The set “Example 1” has 10,000 observations in each class. In set “Example 2”, the majority and minority classes contain 50,400 and 33,600 observations)
Xu et al. [42]	2016	Cross-sectional data	Hyperglycemia with missing covariates using inpatient EHR data from a tertiary care institution affiliated to the University of Florida [*n* = 4,947 observations]; simulated dataset [*n* = 500]
Grundmeier et al. [43]	2015	Cross-sectional data	Pediatric Electronic Health Records [*n* = 170,171]
Bounthavong et al. [44]	2015	Longitudinal data	The Veterans Integrated Systems Network 22 (Desert Pacific Healthcare Network) that includes VA facilities in the Southern California (Los Angeles, Long Beach, Loma Linda, and San Diego) and Nevada (Las Vegas) regions [*n* = 1,400,000]
Vourli et al. [45]	2014	Longitudinal data	The simulated datasets based chronic hepatitis B patients [*n* = 1,000]
Welch et al. [46]	2014	Longitudinal data	The simulated datasets based on THIN data [*n* = 5,000]
Sariyar et al. [47]	2012	Cross-sectional data	German epidemiological cancer registry [*n* = 100,000 records]
Marshall et al. [48]	2010	Longitudinal data	The simulated datasets based on a German breast cancer dataset [*n* = 1,000]

BIFAP, Base de datos para la Investigación Farmacoepidemiológica en Atención Primaria; CPRD, Clinical Practice Research Datalink; DACMI, data analytics challenge on missing data imputation; DFCI, Dana Farber Cancer Institute; EHR, electronic health records; FHS, Framingham Heart Study; MCHS, The Marshfield Clinic Health System; MDC, Multi-Center Diabetic; MIMIC, Medical Information Mart for Intensive Care; MTS, multivariate time series; NHIS, The National Health Interview Survey; NR, not report; PPMI, Parkinson’s progression markers initiative; PRO-ACT; The ALS Pooled Resource Open-access Clinical Trials; THIN; The Health Improvement Network

^a^ The full names of some of the abbreviations for datasets were not found in the original study.

Three common missingness mechanisms were analyzed: MCAR (29/46), MAR (20/46), and MNAR (21/46). Only three articles provided details on missingness patterns, including monotone (*n* = 2) and arbitrary (*n* = 1) patterns [26,31,45]. The percentage of missing data in most datasets (37.0%, 17/46) was 50% or lower, while 15 studies (32.6%) reported missing rates that were equal to or exceed 50%. Furthermore, 29 studies identified missing data as covariates, Two studies as both covariates and outcomes, and 15 studies did not report the type of missing data. A total of 18 studies evaluated the performance of medical statistical methods for missing data, and two focused on machine learning methods (including deep learning and traditional machine learning methods). A total of 26 articles compared the effectiveness of machine learning and medical statistical methods. Multiple imputation by chained equations (MICE; *n* = 22) was found to be a very popular statistical method in medical statistics, while generative adversarial network (GAN)-based methods (*n* = 9) were the most common deep learning approaches. The k-nearest neighbor (KNN) method (*n* = 16) was the most prevalent traditional machine learning method. The mean absolute error (MAE) and root mean square error (RMSE) were used to evaluate the imputation accuracy in these studies. Additionally, 34 studies compared the performance of different methods on downstream tasks (e.g., classification and regression), with a large proportion being machine-learning-based articles (24/34) (see Table 2).

**Table 2.** The missing data addressing methods of 46 included studies

First author /publication year	Type of missing data	Missing data addressing methods			Evaluation indicators	
		Deep learning methods	Traditional machine learning methods	Medical statistical methods	Indicators for the assessment of missing data processing methods	Indicators for the assessment of downstream performance
Weng et al., 2024 [51]	Covariates	MVIIL-GAN; MisGAN; CGAIN; PC-GAIN; MIWAE	KNN; BiScaler	SI; MICE	RMSE; MCC	Classification [AUC]
Psychogyios et al., 2023 [56]	Covariates	DAE with KNN; GAN; GAIN; I-GAIN; NAA; I-NAA	Missforest	Mode/mean imputation; MICE	RMSE	Classification [F1-score]
Zhou et al., 2023 [53]	Covariates and outcomes	None	None	MI; spline smoothing	SE	NR
Sisk et al., 2023 [54]	Covariates	None	None	MI; regression imputations	NR	Regression [calibration in the large; calibration slope; C-statistic; Brier score]
Kazijevs and Samad 2023 [14]	NR	BRITS; CATSI; CATSI-LSTM; LSTM; CATSI-MLP; NAOMI	None	MICE	NRMSD	Classification [true positive; false positive; false negative prediction]
Bernardini et al., 2023 [11]	Covariates	GAN; MisGAN; ccGAN	KNN	MICE	MSE	Classification [accuracy; F1-score; precision; recall; AUC; PRAUC]
Shadbahr et al., 2023 [21]	Covariates	GAIN; Missing data importance-weighted autoencoder	MissForest	Mean; MICE	RMSE; MAE; Coefficient of determination; Kullback–Leibler divergence; Kolmogorov–Smirnov statitstic; Wasserstein distance	Classification [correlation between the discrepancy scores in classes A–C and the performance of the Classification model]
Li et al., 2023 [22]	Covariates	GRU-D; M-RNN; GRMN-SGTM Ensemble; DeepMVI; MVIRA	KNN; HIOC; HIOC+MLP	MICE; SICE; MF	RMSE	Classification [AUROC; AUPRC]
Vader et al., 2023 [23]	Covariates	None	None	MI; propensity score calibration	Bias; MSE	Regression [HR]
Getz et al., 2023 [24]	Covariates	Denoising autoencoders	Random forests	MICE	Bias	Regression [the coefficients of interest based on the proportion of confidence intervals that covered the true value]
Ferri et al., 2023 [25]	Covariates	Translation and encoding; GAN	KNN	Mean; Bayesian ridge regression	NR	Classification [AUC; sensitivity; specificity]
Shen et al., 2022 [57]	Covariates	None	None	Mean; zero imputation; structural equation modeling; MICE	Absolute error	NR
Perez-Lebel et al., 2022 [15]	NR	None	MIA; iterative; iterative+mask; KNN; KNN+mask; iterative+bagging; iterative+mask+bagging; MIA+bagging	Mean; mean+mask; median; median+mask	Deviation of the prediction score	Classification; regression
Samad et al., 2022 [27]	NR	MI with a chain of deep regression models; MI with ensemble learning plus the cluster information	KNN; MI with chains of ensemble learning (gradient boosting and random forest)	MICE; iterative SVD; matrix factorization	NRMSD	Classification [mean; classification accuracy]
Pereira et al., 2022 [26]	NR	PMIVAE; DAE; VAE; GAIN; MIWAE	KNN	Mean; MICE; SoftImp	MAE; RMSE	Classification [F1-score]
Paul et al., 2022 [28]	Covariates	None	None	Two-fold MI; MICE; MI with Monte Carlo Markov Chain	The difference in mean and standard error between imputed versus observed complete case	NR
Mulyadi et al., 2022 [29]	Covariates	1. Unidirectional Models: GRU-D; RITS-I; RITS;V-RIN;V-RIN-full	None	None	MAE; MRE; MSE	Classification [AUC; AUPRC]
		2. Bidirectional Models: M-RNN; BRITS-I; BRITS				
Ouyang et al., 2021 [58]	Covariates	GAIN; M-RNN; BRITS; LGnet; GAN-2-Stage; E^2^GAN; ImputeRNN	KNN	Mean; MICE; matrix factorization	RMSE	Classification [AUC]
Lim et al., 2021 [59]	NR	HIVAE; MIWAE; VAEAC; IMIWAE; NIMIWAE	None	Mean; MICE	Average L1 distance; percent bias	NR
Zhou and Saghapour 2021 [52]	Covariates	GAIN	ImputeEHR1; ImputeEHR2; MissForest; KNNImputer	MICE; SoftImpute	RMSE	Classification [AUC]
Bertsimas et al., 2021 [30]	Covariates	None	OptImpute under KNN objective; Med.KNN	Mean; moving average; Bayesian principal component analysis; MICE; MI with boostrap expectation maximization	MAE; RMSE	Classification [AUC]; Regression [MAE]
Mikalsen et al., 2021 [31]	NR	None	TCK	None	NR	Classification [F1-score; sensitivity; specificity]
Jager et al., 2021 [13]	NR	Discriminative Deep Learning; Generative Deep Learning	KNN; random forest	Mean; mode	RMSE	Classification [F1-score]
Li et al., 2021 [8]	Covariates	None	None	Monotone MI; fully conditional specification; multilevel multivariate missing imputation; multilevel univariate missing imputation	Mean; standard error; and 95% confidence interval of predicted values for each holdout; coverage rate; average width; normalized RMSE	NR
Gwon et al., 2021 [32]	NR	MLP	Self-training regression imputation; random forest; KNN	MICE; mean	Mean squared error; Pearson correlation coefficient	NR
Jazayeri et al., 2020 [60]	NR	None	None	Similarity-based missing data imputation method; MICE; mean; Gaussian processes; 3D-MICE	nRMSD metric	NR
Zhang et al., 2020 [61]	Covariates	Artificial neural network	SVM; random forest	Logistic regression; EM imputation	Prediction score	NR
Leyrat et al., 2020 [55]	Covariates and outcomes	None	None	LOCF; MI; IPMW	Absolute bias	NR
Xu et al., 2020 [33]	NR	Deep autoencoder	Unsupervised method; SoftImpute; KNN; MiceForest	MICE	NRMSE; MAE; RMSE.	Classification [proportion of falsely classified entries; AUC; recall; precision; F-measure]
Venugopalan et al., 2019 [50]	Covariates	None	Robust clustering-based imputation	Mean; EM	NR	Classification [scores accuracy; MCC]
Nagarajan et al., 2019 [34]	NR	None	KNN; KNN based on particle swam optimization; KNN based on genetic algorithms; KNN based on Information gain; KNN based on gray distance; SVM regression imputation; weighted random forest imputation; KNN based on LAHCAWOA	Mean; MI	RMSE	Classification [misclassification error rate]
Hegde et al., 2019 [35]	Covariates	None	None	PPCA; MICE	RMSE	NR
Pham et al., 2019 [36]	Covariates	None	None	SI with the white ethnic group; standard MI; calibrated-adjustment MI	Bias in point estimate; empirical and average model standard errors; coverage of 95% confidence interval	Regression [OR]
Beaulieu-Jones et al., 2018 [49]	Covariates	None	Random forest; KNN	Mean; median; MICE; SVD	RMSE	NR
Yoon et al., 2018 [37]	NR	GAIN; Autoencoder	MissForest	MICE; matrix completion; EM	RMSE	Classification [AUROC]
Martín-Merino et al., 2018 [38]	Covariates	None	None	MI	NR	Cox regression [HR]
Luo et al., 2018 [39]	NR	None	None	MICE; Gaussian process; 3D-MICE	Normalized RMSD; Normalized percentile absolute deviation	NR
Beaulieu-Jones et al., 2017 [40]	NR	Deeply learned autoencoders	KNN	SI; iterative singular value decomposition; SoftImpute	RMSE	Regression [RMSE between the predicted slope and actual slope]
Razzaghi et al., 2016 [41]	NR	None	MLSVM; MLWSVM; SVM; WSVM; C4.5; 5-nearest neighbor	Naive Bayes; logistic regression	NR	Classification [sensitivity; specificity; G-mean; accuracy]
Xu et al., 2016 [42]	Covariates	None	BART	MICE−CART; MICE; Bayesian nonparametric approach	SE	Regression
Grundmeier et al., 2015 [43]	Covariates	None	None	Missing value indicator method; imputation using patient information; imputation with Bayesian improved surname geocoding	NR	Classification; regression [AROC]
Bounthavong et al., 2015 [44]	Covariates	None	None	MI	NR	Regression [OR]
Vourli et al., 2014 [45]	Covariates	None	None	LOCF; MI; IPMW	Estimated relative bias	Regression
Welch et al., 2014 [46]	Covariates	None	None	Two-fold FCS MI algorithm	Empirical variance	Regression
Sariyar et al., 2012 [47]	NR	None	None	Imputation with unique values; sample-based imputation; reduced-model classification	NR	Classification [F-score; precision; recall]
Marshall et al., 2010 [48]	Covariates	None	None	SI; MI methods including:	SE	Regression [regression coefficients]
				1. Data augmentation approach assuming a multivariate normal distribution;		
				2. Data augmentation assuming a general location model;		
				3. Regression switching imputation;		
				4. Regression switching with PMM;		
				5. Flexible additive imputation models		

AUC, Area Under Curve; AUPRC, Area Under the Precision-Recall Curve; AUROC, Area Under the Receiver Operating Characteristic Curve; BART, Bayesian additive Regression trees; BRITS, bidirectional recurrent imputation for time series; CART, Bayesian classification and regression trees; CATSI, context-aware time series imputation; CGAIN, conditional generation adversarial imputation network; ccGAN, clinical conditional generative adversarial network; DAE, denoising autoencoder; DeepMVI, deep learning method for missing value imputation; E^2^GAN, end-to-end generative adversarial network; EM, expectation maximization; FCS, fully conditional specification; GAIN, generative adversarial imputation networks; GAN, generative adversarial network; GRU-D, deep learning models based on gated recurrent unit; GRMV-SGTM, General Regression Neural Network-Successive Geometric Transformation Model; HIVAE, Handling Incomplete Heterogeneous Data using Variational autoencoders; HIOC, hybrid interpolation method combining chained equations; HR, hazard ratio; I-GAIN, improved generative adversarial imputation network; IMIWAE, Ignorably Missing Importance-weighted Autoencoder; IPMW, inverse probability of missingness weighting; I-NAA, improved neighborhood aware autoencoder; KNN, k nearest neighbor classification; LAHCAWOA, local search based feature weighted nearest neighbor imputation; LOCF, last observation carried forward; LSTM, long short-term memory; MAE, mean absolute error; MCC, Matthews correlation coefficient; Med.KNN, MedImpute under KNN objective; MF, matrix factorization; MI, multiple imputation; MIA, Missing incorporated in attributes; MICE, multivariate imputation via chained equations; MIWAE, missing data importance-weighted autoencoder; MLP, multilayer perceptron; MLWSVM, multilevel weighted support vector machine; MRE, mean relative error; MIWAE, missing values importance-weighted autoencoder; M-RNN, multi-directional recurrent neural networks; MSE, mean squared error; MVIRA, model based on missing value imputation and reliability assessment; MVIIL-GAN, missing value imputation and imbalanced learning generative adversarial network; NA, not application; NAA, neighborhood aware autoencoder; NAOMI, non-autoregressive multiresolution imputation; NIMIWAE: Non-Ignorably Missing Importance-weighted Autoencoder; NR, not report; NRMSD, normalized root mean square deviation; OR, odds ratio; PC-GAIN, pseudolabels to train the auxiliary classifier and incorporate it into the GAN; PMIVAE, partial MI with variational autoencoders; PPCA, probabilistic principal component analysis; PMM, predictive mean matching; PRAUC, precision–recall curve; RITS, recurrent imputation for time series; RMSE, root mean square error; SE, square error; SI, single imputation; SICE, single center imputation from multiple chained equation; SVD, singular value decomposition; SVM, support vector machine; TCK, time series cluster kernel; VAE, variational autoencoder; VAEAC, variational autoencoder with arbitrary conditioning; V-RIN, variational-recurrent imputation network.

### Performance of missing value addressing techniques

As indicated in Table 3, we compared the performance differences among medical statistical and machine learning approaches (including deep learning and/or traditional machine learning methods) in 26 research papers. Fourteen studies verified that deep learning methods generally outperform other methods. Eight studies showed that traditional machine learning methods performed better than medical statistical methods, while three papers indicated that they were also superior to deep learning methods. GAN-based methods (*n* = 9) and autoencoders (*n* = 9) were the most prevalent deep learning techniques, whereas KNN-based methods (*n* = 16) and random forests (*n* = 11) were common traditional machine learning methods. Moreover, most methods (12/26, 46.2%) used in longitudinal studies were novel approaches proposed by the authors, including time series cluster kernels, autoencoders, missing value imputation and reliability assessment (MVIRA), MedImpute under the KNN objective (Med.KNN), and clinical conditional generative adversarial network (ccGAN).

**Table 3.** The comparison of performance between machine learning methods and traditional medical statistical methods in addressing missing data. Blank space indicates no report.

First author/publication year)	Study type		Sample size		Percentage of missing data		Missing mechanisms			Missingness methods with superior performance^a^		
	Longitudinal data	Cross-sectional data	≤10,000	>10,000	≤50%	≥50%	MCAR	MAR	MNAR	Deep learning methods	Traditional machine learning methods	Medical statistical methods
Weng et al., 2024 [51]	√		√		√	√				√		
Kazijevs and Samad, 2023 [14]	√		√		√	√	√	√	√	√	NA	
Bernardini et al., 2023 [11]	√		√	√	√	√	√			√		
Shadbahr et al., 2023 [21]	√		√		√		√				√	
Li et al., 2023 [22]	√			√	√	√				√		
Getz et al., 2023 [24]	√		√		√		√	√	√	√		
Ferri et al., 2023 [25]	√			√						√		
Psychogyios et al., 2023 [56]	√	√	√		√		√	√		√		
Perez-Lebel et al., 2022 [15]	√			√						NA	√	
Samad et al., 2022 [27]		√	√	√	√	√	√	√	√	√		
Pereira et al., 2022 [26]			√		√	√			√	√		
Bertsimas et al., 2021 [30]	√		√		√		√		√	NA	√	
Jager et al., 2021 [13]			√	√	√		√	√	√	√	√	√
Gwon et al., 2021 [32]				√	√			√			√	
Zhou et al., 2021 [52]	√		√	√	√	√	√				√	
Ouyang et al., 2021 [58]	√		√	√	√	√			√	√		
Lim et al., 2021 [59]	√		√	√	√	√	√	√	√	√	NA	
Xu et al., 2020 [33]	√		√		√		√			√		
Zhang et al., 2020 [61]	√		√		√							√
Nagarajan et al., 2019 [34]			√		√		√	√	√	NA	√	
Venugopalan et al., 2019 [50]	√			√	√	√		√	√	NA	√	
Yoon et al., 2018 [37]			√	√	√	√	√			√		
Beaulieu-Jones et al., 2018 [49]		√		√	√		√	√	√			√
Beaulieu-Jones et al., 2017 [40]	√		√		√		√		√	√		
Razzaghi et al., 2016 [41]		√		√	√					NA	√	
Xu et al., 2016 [42]		√	√		√		√	√				√

MAR, missing at random; MCAR, missing complete at random; MNAR, missing not at random.

^a^ The “Missingness methods with superior performance” was extracted from the original literature.

### Missingness methods for three missing mechanisms

#### Missing data handling methods for MCAR

Most of the included studies (29/46) considered MCAR mechanisms, where missing data were random and independent of other variables [9]. In these studies, missing values were randomly generated by removing values from a data matrix using the missing data rate. Orthogonally, 17 studies analyzed missing value methods for longitudinal data, and six studies investigated cross-sectional types. One study included datasets from both longitudinal and cross-sectional studies. Five studies did not report the spatiotemporal nature of the data. Twelve studies (41.4%) analyzed only statistical methods, and one (3.4%) analyzed deep learning methods. Another 16 (55.2%) focused on both statistical and machine learning methods (including deep learning and/or traditional machine learning).

For longitudinal datasets, 12 studies had missing values that were equal to or less than 50%. Machine learning methods included deep learning methods like denoising autoencoder (DAE) (*n* = 4) and GAN-based methods (*n* = 3), and traditional machine learning methods like KNN-based methods (*n* = 5) and missForest (*n* = 3). Additionally, MICE (*n* = 7) and single imputation (SI) (*n* = 4) were the most frequently utilized statistical methods. Several deep learning methods, including autoencoders [21], context-aware time-series imputation (CATSI) [14], bidirectional recurrent imputation for time series (BRITS) [14], and ccGAN [11], showed better performance than statistical methods. Traditional machine learning methods such as Med.KNN [30] and other unsupervised methods [33] also performed well. Another study found that the MICE predictive mean matching (MICE-PMM) model outperformed traditional MI [48]. Seven studies with missing data equal to or exceeding 50% compared the performance of deep learning, traditional machine learning, and medical statistical methods. The results indicated that GAN (*n* = 2) and KNN (*n* = 2) were common machine learning missingness approaches while MICE (*n* = 4) was the most common medical statistical method for handling missing values. CATSI [14], BRITS [14], and ccGAN [11] outperformed the MICE model. Two additional studies examined the inverse probability of missingness weighting (IPMW) method [43] and last observation carried forward (LOCF) [55], comparing both to medical statistical methods.

In cross-sectional studies, KNN (*n* = 2) and MICE (*n* = 5) were widely used for missing data equal to or below 50%. Medical statistical methods such as probabilistic principal component analysis (PPCA) [35], Bayesian nonparametric modeling [42], and MICE-based characterization of individual samples with cluster labels (CISCL) [27] performed better than MICE. One study proposed a MICE + CISCL hybrid as a flexible imputation tool for handling missing data equal to or exceeding 50% of the population [27].

#### Missing data handling methods for MAR

MAR assumes that missing data depends on observed values [9]. Twenty studies compared the performance of imputation methods on MAR data. In eight applicable studies, lower or higher marker values implied a greater probability of missing data based on logistic regression. Ten studies focused on missing values in longitudinal data, and five examined cross-sectional datasets. One study included both longitudinal and cross-sectional datasets, and four did not report the spatiotemporal nature of the data. The performance of statistical methods was analyzed in nine studies (45.0%), while 11 studies (55.0%) focused on both medical statistical and machine learning methods (including deep learning and/or traditional machine learning).

Seven studies examined cases with 50% or less missing longitudinal data, and 5 examined cases with more than 50% missing data. Two studies did not report the percentage of missing data. Machine learning strategies included deep learning methods like DAE and imputation methods for time series, and traditional methods like random forests. MI methods (i.e., MICE, data augmentation, regression switching, and PMM), SI, LOCF, and IPMW were common statistical methods. Among these, CATSI [14] and MICE-PMM [48] performed better with **≤**50% missing values, whereas BRITS [14] and IPMW [45] performed well with **≥**50%.

In cross-sectional studies, KNN and deep-learning-based MI methods were common machine learning approaches, while MICE was the most common medical statistical method. Bayesian nonparametric modeling [42] and MICE + CISCL [27] outperformed MICE in cases with **≤**50% missing data and also showed superior performance with ≥50% [27].

#### Missing data handling methods for MNAR

MNAR indicates that missing data are dependent on unobserved values, given the state of observed data [9]. Among the 21 studies focusing on MNAR, 17 discussed methods for generating missing data. Twelve of these studies focused on longitudinal data, four examined cross-sectional data, and five did not report the spatiotemporal state of the data. Eight studies (38.1%) analyzed the performance of medical statistical methods, one (4.8%) incorporated traditional machine learning strategies, and 12 (57.1%) focused on both medical statistical and machine learning methods (including deep learning and/or traditional machine learning).

Eight of the longitudinal datasets had missing values amounting to 50% or less. KNN and DAE were prevalent approaches for addressing missing data in machine learning. Conversely, MI and SI were primarily employed as the statistical methods for managing missing values. In addition, KNN [30], autoencoder [21], BRITS [14], and MICE-PMM [48] outperformed medical statistical methods. Six studies simulated missing data values greater than 50% using a time-series cluster kernel (TCK) [31], IPMW [45], and BRITS [14], with all three methods showing good performance.

Two studies closely examined cross-sectional data and compared the performance of medical statistical methods without identifying an optimal method [43,47]. Other machine learning methods, such as KNN, random forest, and deep-learning-based MI methods, were also reported. Another study indicated that the hybrid MICE + CISCL machine learning model was a good candidate for addressing missing data ranging between 5% and 80% [27].

### Application of software

Nearly a third of the included studies (*n* = 14) used Python, followed by R (*n* = 11), Stata (*n* = 6), and SAS (*n* = 1). Twelve studies did not report the statistical software used. One study used both SAS and R, and another applied both R and Python. Of the 15 studies using Python, all evaluated the performance of machine learning and medical statistical methods. The majority of the studies discussing the use of R (9/13) and all articles discussing Stata (6/6) focused on the impact of medical statistical methods on missing data processing.

## Discussion

Applying inappropriate methods for handling missing data can lead to biased and inaccurate results, which is especially detrimental in EHR studies [24]. As the importance of EHR data in clinical research increases, both medical statistical and machine learning methods for addressing missing data have evolved. This systematic review aims to provide a detailed overview of these methods across various scenarios.

### Missing data scenarios

Ideally, missing data methodologies are tested in realistic scenarios using simulations to compare model performance with ground-truth knowledge. These simulations typically involve four steps [62]: data collection (and model training if a machine learning application is used), manipulating testing data (e.g., masking or removing data items), imputation (i.e., generating missing values), and computing model accuracy. Evaluating the inference ability of the imputation method determines its effectiveness. It is crucial to ascertain the context, purpose, and scenario in which data imputation is needed to select the most appropriate method. The 46 reports included in this study focused on medical statistical methods, machine learning models, or combinations of both. Consequently, simulation procedures varied widely among these studies. For instance, the majority of studies (9/10) involving machine learning models applied linear methods to generate missing data for the MNAR mechanism, while studies using medical statistical methods (5/7) consistently reported logistic regression.

Our results indicated that MCAR was the most common data state requiring imputation (63.0%, 29/46), particularly in studies comparing machine learning methods. However, MCAR rarely occurs in reality [8,35,44,46]. One reason for this trend is that MCAR provides a discreetly testable case, where determining model accuracy is a straightforward process [9] and simple simulations suffice [23,36,45,47,48]. Furthermore, EHR data have specific peculiarities not captured by any pre-specified design, making accurate assessment of the missing mechanism relatively complex. MAR and MNAR are more likely to occur in studies using EHR data compared with MCAR.

Three of the reviewed articles reported missingness patterns. In outpatient care, patients intermittently miss scheduled visits, resulting in arbitrary missing data, whereas monotone missingness occurs when patients discontinue visits after a certain point [63]. Thus, identifying the missing data pattern type is crucial when considering imputation methods, especially in longitudinal studies [64].

Regarding the percentage of missing values, studies comparing machine learning methods typically simulated a higher rate of missing data compared to those reporting medical statistical methods. In this review, 15 studies simulated missing data percentages greater than 50%, and those that poorly configured simulations had percentages above 50% [14,23,26,27,46,48]. Therefore, additional research is needed to validate the indispensability and computational accuracy of data processing involving a greater proportion (≥50%) of missing data.

### Opportunities and challenges for missing data handling strategies

The existing statistical literature offers a comprehensive framework for addressing missing data. Our review of 46 studies revealed the identification of up to 15 distinct methods for handling missing data, indicating an increased emphasis on addressing this issue in EHRs. Regarding conventional medical statistical techniques, imputation-based methods (89.1%), particularly MI (76.1%), remained prevalent for handling missing data. MI can create complete data by estimating missing values from an original and incomplete dataset, offering advantages in terms of efficiency and validity [65]. The MICE model is the most popular approach for addressing missing data (54.3%) [66]. This model allows for the imputation of multiple variables, accommodates clustered data, and does not require the specification of joint variable distributions [67]. However, the MICE model relies on the MAR assumption, which may not always hold for clinical data, limiting its application scope. Additionally, non-imputation-based methods like IPMW and Bayesian nonparametric methods have been proposed to mitigate methodological statistical drawbacks. For example, the IPMW method outperformed medical statistical methods, including those facing MNAR scenarios.

The most prominent characteristics of medical statistical imputation methods are model-driven (e.g., regression model and probabilistic model) and require a strict hypothetical proposition based on the missingness mechanism. In reality, this task is extremely difficult [68]. Standard methods for MI can either fail to capture nonlinear relationships or face issues of incompatibility and incongruity when applied to EHR data characterized by high dimensionality and different variable types. However, generative machine learning models are data-driven and do not require identification of the missing data mechanism, data distribution, or learning representation.

Our results showed that both deep learning and traditional machine learning methods (i.e., KNN, GAN, DAE, and random forest) consistently outperformed medical statistical methods in the included studies. Remarkably, deep learning methods often outperformed traditional machine learning methods, which aligns with previous studies, because these methods are tailored to consider data type and missing patterns, thereby improving the quality of data imputation [18]. These data-driven machine learning methods are currently receiving increased attention in research on tasks involving missing EHRs. Among the studies included in this review, a majority of recently proposed methodologies for handling missing data involve machine learning techniques. Notably, few medical statistical methods consider temporal patterns in EHRs to impute missing values. Longitudinal studies often utilize unique machine learning strategies for addressing missing data, such as imputation methods grounded in time-series analysis, GAN-based methods, and DAEs.

Regarding missing value imputation methods in the included studies, they can be broadly classified into parametric imputation (e.g., linear regression) and non-parametric imputation (e.g., nearest neighbor method or non-parametric regression imputation) [69]. Parametric regression imputation requires correctly specifying a parametric model for the dataset. However, in EHR datasets, it is often impossible to determine the distribution that accurately captures attribute correlations within real data. Therefore, non-parametric techniques, widely used in machine learning and pattern recognition, are effective for handling EHR missing data due to their computational efficiency, robustness, and stability. This trend is evident in several studies included in this literature review, such as TCK-based methods and autoencoders. Given the unique characteristics of non-parametric machine learning methods, advanced approaches are necessary to ensure model generalizability and robustness across different datasets through retraining.

The algorithms and procedures used to compute missingness in EHR data are usually more complex than those used in clinical trials or observational studies. As such, addressing missing data should be based on the interrelationships among various variables, taking into account relevant factors such as missing mechanisms and missing patterns. For example, the computing procedures of imputation-based algorithms are based on cohort information or calculated individually for each patient, relying on inter-attribute correlations. There are several types of correlations, including cross-sectional (involving all samples) and longitudinal (involving each individual), or univariate (within a single variable) and multivariate (involving multiple variables) [8]. In addition, the algorithms for imputing missing values are also related to the sample size of datasets. A previous study illustrated that incorporating appropriate auxiliary variables in the imputation model can alleviate MAR- or MNAR-related bias. However, this practice may also lead to a significant increase in the standard errors of the estimates, particularly in cases where the sample size is limited [8].

Machine learning approaches for incremental optimization have several advantages. For example, multiple data sources can be integrated to improve imputation accuracy and dynamic adaptation. They also face challenges. Owing to the complexity and opacity (black box) of these models, the results often cannot be explained despite their accuracy. Hence, there is a notable lack of trust in medical fields, as clinical trials require discrete causative reports. Therefore, a trade-off between model performance and complexity is needed to ensure the reliability of medical decision support tools. As EHRs are generated from many different sources and health information systems, data heterogeneity increases the difficulty of establishing a universal imputation method that is both statistical and learning-based. This phenomenon was evidenced by our observation of various imputation performance results based on different data sources [15]. Future studies should examine comprehensive missingness mechanisms, generalizable patterns, and ubiquitous variables.

Our study has several limitations. In light of the constraints imposed by the available studies, our analysis focused solely on four influential factors pertaining to the establishment of missing data scenarios. However, the effects of the types of missing variables (i.e., continuous variables and categorical variables) and missing patterns in longitudinal datasets (i.e., monotone and arbitrary types) on the performance of different missing data handling methods have not been extensively explored. In addition, the heterogeneity of the included literature incorporated in the study is extensive, encompassing various databases, evaluation of missing data handling methodologies, and assessment criteria. The relative performance of the approaches varies across datasets; thus, we found that none of the missing data strategies were perceived to be one-size-fits-all. The lack of standardized data may result in model performance instability when dealing with different data sources. More evidence related to a benchmark study considering comprehensive missingness scenarios needs to be generated to support these studies. Furthermore, according to previous evidence, the line between machine learning and statistics is at best blurry. There remain certain missing data handling techniques whose classification as either traditional statistical methods or machine learning algorithms is indeterminate, such as MICE and KNN. To enhance the clarity of the summary, we have organized and classified the methods utilized in the included literature to align as closely as possible with previously published research. Finally, due to the focus on evaluating missing data handling strategies in EHRs and the absence of corresponding quality assessment tools available, we did not analyze the research quality of the included studies or register in a publicly accessible database.

### Conclusion

Machine learning methods provide promising opportunities to handle missing EHR data, particularly in longitudinal studies. Considering the heterogeneity of the reviewed studies, none of the missing data methods were considered to be fully generalizable. A broad benchmark study with integrated missingness scenarios that considers missingness mechanisms, missingness patterns, and percentages of missing values is needed in the near future.

## Data Availability

The data supporting this study’s findings are available from the corresponding authors upon reasonable request.

## Supplementary Materials

Supplementary 1 Supplementary Materials 1: PRISMA 2020 Checklist Supplementary Materials 2: Search Strategy
